# Clinical Validation of DNA Methylation Detection in Cervical Exfoliated Cells for Endometrial Cancer in Women with Suspected Lesions

**DOI:** 10.3390/diagnostics16020174

**Published:** 2026-01-06

**Authors:** Yi Yu, Tingting Su, Hongwei Zhang, Qing Li, Qing Cong, Long Sui, Limei Chen

**Affiliations:** 1 Hysteroscopy Center, Obstetrics & Gynecology Hospital of Fudan University, Shanghai 200433, China; icefishyuyi@163.com (Y.Y.);; 2 Department of Cervical Diseases, Qingpu Branch of Obstetrics & Gynecology Hospital of Fudan University, Shanghai 200437, China; 3 Shanghai Key Lab of Female Reproductive Endocrine Related Diseases, Obstetrics & Gynaecology Hospital of Fudan University, Shanghai 200433, China

**Keywords:** endometrial cancer, CDO1/CELF4 methylation, early detection, diagnostic adjunct, exfoliative cervical cytology, transvaginal sonography

## Abstract

**Background/Objectives:** Currently, no non-invasive detection method for endometrial cancer (EC) is recommended in clinical practice worldwide. This study aimed to evaluate the clinical value of detecting DNA methylation of CDO1 and CELF4 (CDO1m/CELF4m) in exfoliated cervical cells for the detection of EC in women with suspected endometrial lesions. **Methods:** A total of 2164 patients scheduled for hysteroscopic surgery due to suspected endometrial lesions at the Obstetrics and Gynecology Hospital of Fudan University between July 2023 and May 2024 were prospectively enrolled. Preoperative exfoliated cervical cells were collected for dual-gene methylation testing. Clinical data and endometrial thickness measured by transvaginal sonography (TVS) were recorded. Hysteroscopic histopathological diagnosis served as the gold standard to evaluate the performance of methylation testing alone and in combination with TVS. **Results:** This study included 2164 patients, comprising 33 EC cases, 31 cases of endometrial intraepithelial neoplasia (EIN), and 2100 cases of non-endometrial lesions, with mean ages of 51.7 ± 6.4, 49.5 ± 8.9, and 44.7 ± 9.8 years, respectively (*p* < 0.001). For EC detection, CDO1m/CELF4m positivity showed a sensitivity of 93.94% (95% CI: 79.77–99.26%), specificity of 96.7% (95% CI: 95.92–97.47%), positive predictive value (PPV) of 31.0% (95% CI: 25.96–36.53%), and negative predictive value (NPV) of 99.90% (95% CI: 99.63–99.98%). For EIN detection, the sensitivity was 83.87%, specificity 97.95%, PPV 37.68%, and NPV 99.76%. Combining TVS with DNA methylation detection further improved the sensitivity and NPV for both EC and EIN detection. **Conclusions:** DNA methylation detection in exfoliated cervical cells demonstrates high sensitivity and specificity for EC detection. The combination with TVS further enhances sensitivity and NPV, offering a simple and non-invasive triage strategy for patients with suspected endometrial lesions. This study was registered in China Clinical Trial Registry (ChiCTR2200055991) on 30 January 2023.

## 1. Introduction

Endometrial cancer (EC) is the most common malignant tumor of the female lower reproductive tract. According to data released by the National Cancer Clinical Research Center of China in 2022, there were 84,500 new EC cases and 17,500 deaths, surpassing the figures reported in the United States during the same period [1,2]. The incidence of EC is rising, driven by societal development and increasing trends in obesity and longevity. EC is traditionally classified into two histopathological types [3]. Type I EC, also known as “estrogen-dependent” EC, accounts for 80% of cases, typically affecting younger women and often presenting with abnormal vaginal bleeding. Type II EC is “non-estrogen-dependent,” frequently occurring in thin, elderly women without obvious symptoms like bleeding or endometrial thickening [4,5]. The prognosis of type I EC is generally fair, whereas type II EC is usually diagnosed at an advanced stage, leading to poor outcomes. Consequently, the early identification of high-risk patients and proactive detection for cancer and precancerous lesions are crucial for improving EC prognosis.

EC diagnosis primarily relies on invasive procedures such as diagnostic curettage, hysteroscopy, and endometrial aspiration biopsy to obtain pathological tissue [6,7]. Currently, no non-invasive detection method is recommended for clinical practice worldwide. TVS is the preferred imaging modality for evaluating endometrial lesions, but its sensitivity and specificity are suboptimal [8]. However, its performance varies across patient subgroups. Diagnostic thresholds are less specific in premenopausal women, and sensitivity can be limited in obese patients. Its low specificity is particularly problematic in women presenting with abnormal uterine bleeding (AUB), where an accurate triage tool is needed to avoid unnecessary invasive procedures. Consequently, there is an urgent clinical need for improved detection methods.

Recent advancements in the molecular and genomic profiling of EC, including the role of L1CAM as a predictor of poor prognosis [9], have been notable. Epigenetic silencing of tumor suppressor genes plays a crucial role in tumor initiation and progression [10,11]. As one such epigenetic modification, DNA methylation can be detected in various tumor tissues (precancerous lesions, early and advanced lesions) or in different body fluids and cells from patients with cancer [12,13]. Consequently, DNA methylation is widely utilized as a novel biomarker for early detection of various malignant tumors. Numerous studies have explored methylation in EC. Recent research indicates that self-collection of exfoliated cervical cells can improve early EC diagnosis, leading to several clinical trials investigating cervical cytology methylation detection for EC.

Based on these findings, this study employed CDO1 methylation (CDO1m) and CELF4 methylation (CELF4m) detection kits to analyze exfoliated cervical cells, aiming to detect EC in women with suspected endometrial lesions and evaluate the accuracy of cytological DNA methylation screening, both alone and in combination with TVS, for diagnosing EC.

## 2. Materials and Methods

### 2.1. Research Materials

This prospective clinical study was conducted at the Hysteroscopy Center of Obstetrics and Gynecology Hospital, Fudan University. The study protocol was prospectively registered with the Chinese Clinical Trial Registry (No. ChiCTR2200055991) on 30 January 2023, prior to participant enrollment. It was approved by the Ethics Committee of Fudan University Obstetrics and Gynecology Hospital (Approval number: 2023-42; approval date: 15 May 2023). Informed consent was obtained from all subjects involved in the study.

Inclusion Criteria: Participants were required to (A) provide an exfoliated cervical cell sample; (B) have at least one EC risk factor (e.g., endocrine disorder, obesity, hypertension, diabetes, early menarche, late menopause, infertility, ovarian cysts, exogenous estrogen use, or genetic predisposition) per clinical guidelines; and (C) provide written informed consent.

Exclusion Criteria: Samples were excluded if (1) histologically confirmed EC had been surgically removed prior to sampling; (2) radiotherapy/chemotherapy for any cancer was received within the preceding 3–6 months; (3) CIN2+ or cervical cancer was diagnosed; (4) collection/storage deviated from kit instructions; (5) contamination was suspected; or (6) clinical information was missing or incomplete.

### 2.2. Clinical Trial Flow Chart

**Figure 1:**
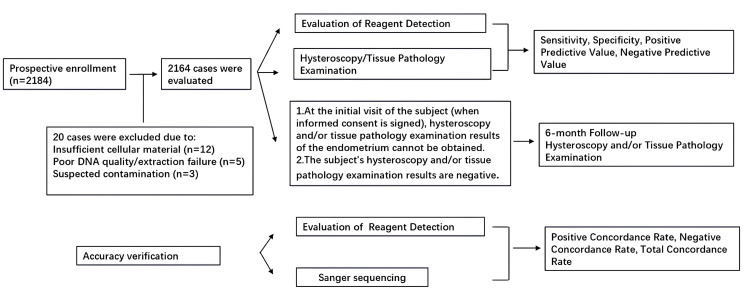
The flow chart of clinical study.

Exfoliated cervical cells were collected preoperatively for dual-gene methylation detection. Basic clinical data, including TVS-measured endometrial thickness, were gathered. The sensitivity, specificity, positive predictive value (PPV) and negative predictive value (NPV) of the methylation detection for endometrial detection were evaluated using hysteroscopic histopathological diagnosis as the gold standard. A detailed participant flow diagram is provided in Figure 1, summarizing the number of women assessed for eligibility, reasons for exclusion, and the final cohorts included in the methylation and histopathological analyses.

### 2.3. Collection of Clinical Data

General data collected from the enrolled patients included age, menstrual history (including uterine bleeding or postmenopausal vaginal bleeding), gravidity, medical history, body mass index (BMI), endometrial thickness of TVS within one month prior to enrollment, and presence of polycystic ovary syndrome (PCOS). In this study, a positive TVS finding was defined as an endometrial thickness > 11 mm in premenopausal women, >5 mm in postmenopausal women, or the presence of an intracavitary mass or irregular endometrial echo. In this study, TVS was used as an auxiliary assessment tool for preliminary evaluation of endometrial status in patients with suspected lesions (not as an EC screening tool for asymptomatic women). The combined positivity rule was defined as a positive result if either TVS or CDO1m/CELF4m testing was positive, prioritizing the reduction in missed diagnoses for EC/EIN.

### 2.4. DNA Extraction and Methylation Detection of Exfoliated Cervical Cells

Clinical trial specimens were derived from exfoliated cervical cell samples. Methylation detection was performed in a certified DNA laboratory by personnel blinded to the patients’ clinical information, TVS, hysteroscopy, and histopathology results. The Human CDO1 and CELF4 Gene Methylation Detection Kits (PCR-fluorescent probe method; Item No. 20220038, Beijing Qiyuan Juhe Biotechnology Co., Ltd., Beijing, China) were used, with reagents stored at −20 ± 5 °C protected from light.

### 2.5. Sample Collection and Detection Process

Samples were assessed for adequacy based on cellular content and DNA quality. Inadequate samples (*n* = 20) were excluded prior to methylation testing and not included in the final cohort. No repeat sampling was performed. All statistical analyses of test performance were based on samples with valid methylation and histopathological results.

After obtaining informed consent, cervical cytology samples were collected before hysteroscopy. Cervical surface mucus was gently wiped away with a sterile cotton ball. A BD liquid-based cytology brush was inserted approximately 1 cm into the cervical canal, rotated five to six times in one direction, and then the brush head was placed into the BD preservative solution. Following cell centrifugation, cervical cell DNA was extracted using a DNA extraction kit. The extracted DNA was then bisulfite-converted. The resulting bisulfite-treated DNA (bis-DNA) was immediately subjected to PCR amplification according to the kit instructions (NCT05290922). PCR was performed on an ABI 7500 real-time PCR system under the following conditions: pre-denaturation at 96 °C for 10 min; 45 cycles of denaturation at 94 °C for 15 s, annealing at 64 °C for 5 s, and extension/fluorescence collection at 60 °C for 30 s; final cooling at 25 °C for 1 min. The ΔCt was calculated as Ct (target gene) − Ct (reference gene). The Youden index was used to determine the positive methylation threshold.

### 2.6. Statistical Methods

Data were recorded in Excel and analyzed using SPSS 20.0. Quantitative data are presented as mean ± standard deviation and compared using the *t*-test. Categorical data are expressed as percentages (%) and compared using the χ^2^ test. Statistical significance was set at *p* < 0.05. The sensitivity, specificity, PPV, NPV, and their 95% confidence intervals (CIs) for methylation detection in detecting EC, EIN, and non-endometrial lesions were calculated. The ΔCt cut-off for methylation positivity was determined using the Youden index to maximize overall diagnostic accuracy. Due to the limited number of endpoint cases, internal cross-validation was not performed; the threshold, therefore, requires external validation. The reporting of this study adheres to the Standards for Reporting Diagnostic Accuracy Studies (STARD) 2015 statement [14], ensuring comprehensive disclosure of its design, conduct, and analysis.

## 3. Results

### 3.1. General Conditions and Pathological Results of Patients

This study successfully included 2164 patients: 33 (1.5%, 95% CI: 1.1–2.2%) in the endometrial cancer (EC) group, 31 (1.4%, 95% CI: 1.0–2.1%) in the endometrial intraepithelial neoplasia (EIN) group, and 2100 (97.1%, 95% CI: 96.3–97.7%) in the non-endometrial disease group. Mean ages were 51.7 ± 6.4, 49.5 ± 8.9, and 44.7 ± 9.8 years, respectively, showing significant differences among the three groups (*p* < 0.001). Age did not differ significantly between the EC and EIN groups (*p* = 1.000) but was significantly different between the EC and non-lesion groups (*p* < 0.001). Significant differences were observed in age, BMI, abnormal/postmenopausal bleeding, and gene methylation status between the EC and non-endometrial disease groups (*p* < 0.001). However, clinical characteristics did not differ significantly between the EC and EIN groups (*p* > 0.05). The results identified abnormal uterine bleeding/postmenopausal bleeding, endometrial thickening (premenopausal > 11 mm or postmenopausal > 5 mm), PCOS, diabetes, and obesity as high-risk factors for EC, shared by both EC and EIN patients. Clinical characteristics and comparisons are detailed in Table 1.

### 3.2. Clinical Efficacy Analysis of EC Detection Indicators

There were 31 cases positive for methylation among 33 cases of endometrial cancer, 26 cases positive in 31 cases of EIN, and 114 cases positive in 2100 cases of non-endometrial lesions. The sensitivity, specificity, PPV, and NPV of CDO1m/CELF4m positivity for predicting EC were 93.94% (95% CI: 79.77–99.26%), 96.7% (95% CI: 95.92–97.47%), 31.0% (95% CI: 25.96–36.53%), and 99.90% (95% CI: 99.63–99.98%), respectively. Both sensitivity and specificity were notably high. Combining methylation testing with TVS improved sensitivity and NPV to 100% (95% CI: 87.02–100%) and 100% (95% CI: 99.68–100%), respectively (Table 2). This study temporarily did not conduct statistical validation of incremental advantage (e.g., NRI, DCA) due to the low prevalence of EC in the single-center cohort (1.5%), which may affect statistical power. Future multi-center studies with expanded sample sizes will supplement these analyses.

Among the 33 histologically confirmed EC cases, the distribution according to the International Federation of Gynecology and Obstetrics (FIGO) 2009 staging system was as follows: Stage IA (*n* = 18, 54.5%), Stage IB (*n* = 10, 30.3%), Stage II (*n* = 2, 6.1%), Stage IIIA (*n* = 2, 6.1%), and Stage IIIC (*n* = 1, 3.0%). Thus, 28 cases (84.8%) were diagnosed at an early stage (Stage I). Methylation positivity was observed in 26 of the 28 Stage I cases (92.9%) and in all 5 of the higher-stage cases (100%).

### 3.3. Clinical Efficacy Analysis of EIN Screening Indicators

For EIN prediction, CDO1m/CELF4m positivity showed a sensitivity of 83.87% (95% CI: 66.27–94.55%), specificity of 97.95% (95% CI: 97.25–98.51%), PPV of 37.68% (95% CI: 30.22–45.77%), and NPV of 99.76% (95% CI: 99.46–99.89%), respectively. The sensitivity, specificity, and negative predictive value were all notably high. When combined with TVS, sensitivity, specificity, and NPV increased to 93.55% (95% CI: 77.16–98.87%), 98.10% (95% CI: 97.39–98.62%), and 99.90% (95% CI: 99.61–99.98%), respectively (Table 3).

### 3.4. Analytical Results of Reagent Detection Accuracy

To verify the accuracy of methylation detection for each gene locus, 205 samples were randomly selected from the enrolled participants and sent to a third-party testing company for analysis using Sanger sequencing. The positive coincidence rates for both CDO1 and CELF4 were 100% (95% CI: 96.90–100%, 97.44–100%, respectively), with Kappa values of 0.9900 and 0.9885, respectively, indicating almost perfect agreement (Table 4).

### 3.5. Performance of Methylation Detection Stratified by Menopausal Status

To evaluate the assay’s applicability across different patient groups, performance was analyzed based on menopausal status. Among the 2164 patients, 1324 were premenopausal and 840 were postmenopausal. For EC detection, sensitivity was 92.3% (12/13, 95% CI: 64.0–99.8%) in premenopausal women and 95.0% (19/20, 95% CI: 75.1–99.9%) in postmenopausal women. Specificity was 96.6% (1266/1311) and 96.8% (794/820) in the two groups, respectively. For EIN detection, sensitivity was 84.6% (11/13) in premenopausal and 83.3% (15/18) in postmenopausal women, with specificities of 97.9% and 98.0%, respectively.

## 4. Discussion

With the widespread use of cervical cytology smears in cervical cancer screening, the diagnostic rate of early cervical cancer and its precancerous lesions has greatly improved, leading to a substantial reduction in cervical cancer mortality rates. However, early detection for endometrial and ovarian cancers remains challenging [15]. Current detection methods primarily depend on invasive operations like endometrial biopsy or laparoscopy, limiting large-scale population screening feasibility [16].

This study is among the first to prospectively evaluate the use of DNA methylation markers (CDO1/CELF4) in cervical exfoliated cells for detecting EC and EIN in a large cohort of patients with suspected endometrial lesions undergoing hysteroscopy. Our findings confirm that this non-invasive method, particularly when combined with TVS, significantly improves diagnostic sensitivity for EC and precancerous lesions, underscoring its clinical potential.

The selection of DNA methylation detection of exfoliated cervical cells for the screening and diagnosis of endometrial cancer is an important innovation in this study. DNA methylation is a stable epigenetic marker with great promise for non-invasive early cancer diagnosis [17,18], as these changes can be detected in early tumor tissues and various biofluids [12,13]. Unlike blood-based ctDNA, which may lack sensitivity for early-stage EC, especially non-aggressive types [19], cervical cells offer direct access to the uterocervical environment. While prior studies have used cervical samples for cytology [20], endometrial cancer target gene detection [21], genetic syndrome diagnosis [22], exosome analysis [23], MSI detection [24], and multi-omics detection [25], robust epigenetic screening protocols for clinical use are lacking.

The use of exfoliated cervical cells for endometrial cancer detection has several significant advantages: (1) It is completely non-invasive. For women who are sexually active, sampling does not cause any damage to the vagina or cervix, making it simpler and safer than retaining peripheral blood. (2) It is easy to obtain, and the quantity of cells is sufficient. A small amount of vaginal washing fluid is adequate for methylation detection and is highly consistent with histological results [25]. (3) It reduces the economic burden on patients. It can be used with the same specimen for cervical cancer cytology screening, and 2 mL of cervical cytology samples is sufficient for testing, thereby reducing the expenditure on specimens and consumables.

The use of exfoliated cervical cytology for endometrial cancer detection requires reliable target genes to differentiate tumor origin [26]. CDO1 and CELF4, often epigenetically silenced in EC but rarely in normal tissues, are promising biomarkers. While prior research proposed a panel (BHLHE22, CDO1, CELF4) for EC diagnosis [27,28], our study prospectively validates CDO1/CELF4 in a large clinical cohort. Compared to the earlier three-gene panel, our dual-gene assay demonstrated comparable sensitivity (93.94% vs. 90.9%) and higher specificity (96.7% vs. 91.4%). Furthermore, our findings highlight exceptional sensitivity for Type II EC (100%) and show that combining methylation testing with TVS achieves 100% NPV, enhancing its utility for clinical triage.

TVS has emerged as the preferred detection method for the clinical diagnosis of patients with suspected endometrial lesions due to its advantages of being non-invasive, cost-effective, and convenient [29]. Notably, our study population is patients with suspected endometrial lesions (not asymptomatic individuals), so TVS here functions as an auxiliary assessment tool rather than a population-based screening tool—consistent with its clinical application positioning in guidelines. Although it demonstrates high diagnostic sensitivity, it suffers from low specificity, often resulting in unnecessary invasive operations. In this study, cytological methylation detection combined with TVS further improved the sensitivity of detecting endometrial cancer and precancerous lesions. However, owing to the low specificity of TVS, combined detection did not achieve a significantly higher level of specificity.

Notably, the high NPV (>99%) supports a clinical “rule-out” function, helping to avoid unnecessary invasive procedures in negative cases. The modest PPV reinforces that a positive result requires further diagnostic confirmation, positioning the test as a triage tool rather than a standalone diagnostic test. The high NPV observed here is partly related to the low prevalence of EC/EIN in our cohort. Clinically, a high NPV helps exclude disease in negative cases and may reduce overtreatment. The modest PPV reflects the large number of non-lesion participants; future studies in higher-risk populations should prioritize monitoring PPV and false-positive rates, and consider combining methylation testing with imaging or clinical risk stratification to enhance clinical utility.

We analyzed methylation and ultrasound findings by EC subtype. Of 33 EC cases, 27 were Type I (25 methylation-positive, 2 negative; 10 with ultrasound abnormalities) and 6 were Type II (all methylation-positive; 1 with ultrasound abnormalities). For Type I EC, methylation detection had a sensitivity of 92.59%, specificity of 96.76%, PPV of 26.60%, and NPV of 99.90%; for Type II EC, these metrics were 100.00%, 96.76%, 8.00%, and 100.00%, respectively—demonstrating higher sensitivity for Type II EC. Given Type II EC’s atypical clinical manifestations, methylation testing’s high sensitivity and NPV can improve its detection rate and reduce missed diagnoses, with high clinical value. This is biologically plausible: Type II EC is driven by molecular pathways rich in epigenetic alterations, including promoter hypermethylation of tumor suppressor genes like CDO1 and CELF4 [27]. However, Type II EC’s small sample size warrants further multi-center validation.

These findings support three key clinical roles: A reliable rule-out tool for intermediate-risk women (e.g., AUB with unclear ultrasound), avoiding unnecessary invasive procedures; Complementary to inconclusive TVS; Prioritizing hysteroscopy in resource-limited/high-volume settings by combining with TVS, optimizing resource use. Integrating this non-invasive triage can enhance patient stratification and diagnostic efficiency.

The most innovative aspect of this study lies in applying cervical exfoliated cell methylation detection for EC detection, which is a non-invasive examination compared to endometrial aspiration and hysteroscopy. Furthermore, our study features a larger sample size and broader population coverage than many previous studies, aligning well with the clinical need to screen high-risk individuals.

Several limitations warrant acknowledgment. The single-center design and the relatively small number of EC/EIN cases limit the generalizability and statistical stability of our estimates, including the derived ΔCt cut-off. Furthermore, our study population consisted of women already referred for hysteroscopy due to suspected lesions, which introduces a high-risk selection bias and limits direct extrapolation of our findings to asymptomatic, average-risk screening populations. While we collected key clinical parameters, the limited number of endpoint cases precluded a robust multivariable analysis to assess the independent diagnostic contribution of methylation after adjusting for potential confounders. The absence of long-term follow-up also restricts insight into the longitudinal stability of methylation markers and the outcomes of methylation-negative cases—information critical for defining optimal screening intervals. Notwithstanding these constraints, the DNA methylation assay presented herein offers a viable and patient-friendly detection option for the triage of symptomatic women.

## 5. Conclusions

In summary, the analysis of our large single-center cohort demonstrates that DNA methylation testing in cervical exfoliated cells exhibits high sensitivity and specificity for detecting EC and EIN. When combined with TVS, sensitivity and NPV are further enhanced, offering a promising, non-invasive triage strategy for patients with suspected endometrial lesions. However, these promising results should be interpreted cautiously due to the limited number of disease cases, the enriched symptomatic cohort, and the modest positive predictive value. Therefore, before this approach can be suggested as a population-based screening tool, larger multicenter trials are required, particularly in asymptomatic or screening-eligible populations, to validate its performance, reproducibility, and clinical utility in broader settings.

## Figures and Tables

**Table 1:** Comparison of general clinical features among the endometrial cancer (EC), endometrial intraepithelial neoplasia (EIN), and non-endometrial lesion groups.

Parameter, Mean ± SD or *n*	Endometrial Cancer Group a (*n* = 33)	Endometrial Pre-Cancerous Lesion Group b (*n* = 31)	Non-Endometrial Pre-Cancerous Lesion Group c (*n* = 2100)	abc Groups Statistical Values F Value	P_abc_=	P_ab_=	P_ac_=	P_bc_=
Age	51.7 ± 6.4	49.5 ± 8.9	44.7 ± 9.8	11.97	<0.001	1.000	<0.001	0.02
BMI (kg/m^2^)	26.5 ± 2.2	25.9 ± 2.7	23.2 ± 2.1	60.06	<0.001	0.693	<0.001	<0.001
BMI ≥ 25	24 (72.73%)	20 (64.51%)	613 (29.2%)	46.480	<0.001	0.592	<0.001	<0.001
Diabetes	6 (18.2%)	6 (19.4%)	196 (9.3%)	6.364	0.041	1.000	0.122	0.066
PCOS	8 (24.2%)	8 (25.8%)	208 (9.9%)	16.901	<0.001	1.000	0.014	0.01
AUB	28 (84.8%)	22 (71.0%)	898 (42.8%)	32.802	<0.001	0.149	<0.001	0.002
Endometrial thickening	11 (33.3%)	15 (48.4%)	423 (20.1%)	18.048	<0.001	0.166	0.055	<0.001
methylation-positive	31 (93.9%)	26 (83.9%)	114 (5.4%)	599.149	<0.001	0.188	<0.001	<0.001

**Table 2:** Clinical efficacy analysis of detection indicators for endometrial cancer (EC) (95% CI).

	Sensitivity (%)	Specificity (%)	Positive Predictive Value (%)	Negative Predictive Value (%)
CDO1m/CELF4m (+)	93.94 (79.77–99.26)	96.7 (95.92–97.47)	31.0 (25.96–36.53)	99.90 (99.63–99.98)
CDO1m (+)	83.87 (65.53–93.91)	96.53 (95.64–97.25)	26.00 (17.97–35.90)	99.76 (99.40–99.91)
CELF4m (+)	90.91 (74.53–97.62)	96.72 (95.84–97.41)	30.00 (21.45–40.11)	99.85 (99.54–99.96)
Endometrial thickening/heterogeneity	50.0 (28.22–71.78)	78.60 (76.73–80.39)	2.53 (1.67–3.83)	99.30 (98.93–99.54)
AUB/Postmenopausal bleeding	69.70 (51.13–83.79)	62.62 (60.50–64.69)	2.85 (1.85–4.31)	99.25 (98.57–99.62)
TVS + CDO1m/CELF4m (+)	100 (87.02–100)	75.72 (73.76–77.58)	6.43 (4.53–9.01)	100 (99.68–100)

**Table 3:** Clinical efficacy analysis of detection indicators for endometrial intraepithelial neoplasia (EIN) (95% CI).

	Sensitivity (%)	Specificity (%)	Positive Predictive Value (%)	Negative Predictive Value (%)
CDO1m/CELF4m (+)	83.87 (66.27–94.55)	97.95 (97.25–98.51)	37.68 (30.22–45.77)	99.76 (99.46–99.89)
CDO1m (+)	70.97 (51.76–85.11)	97.76 (97.01–98.33)	31.88 (21.47–44.33)	99.56 (99.14–99.79)
CELF4m (+)	77.42 (58.46–89.72)	97.86 (97.12–98.42)	34.78 (23.98–47.29)	99.66 (99.27–99.85)
Endometrial thickening/heterogeneity	51.72 (32.53–70.55)	79.06 (77.18–80.84)	3.55 (2.50–5.02)	99.10 (98.69–99.38)
AUB/Postmenopausal bleeding	64.52 (45.38–80.17)	62.62 (60.50–64.69)	2.48 (1.57–3.88)	99.17 (98.47–99.56)
TVS + CDO1m/CELF4m (+)	93.55 (77.16–98.87)	98.10 (97.39–98.62)	42.03 (30.45–54.50)	99.90 (99.61–99.98)

**Table 4:** Accuracy analysis of methylation reagent detection.

	Positive Coincidence Rate (%)	Negative Coincidence Rate (%)	Total Compliance Rate (%)	Kappa Value
CDO1	100 (96.90–100)	98.86 (93.83–99.97)	99.51 (97.31–99.99)	0.9900 (0.9705–1)
CELF4	100 (97.44–100)	98.41 (91.47–99.96)	99.51 (97.31–99.99)	0.9885 (0.9660–1)

## Data Availability

The data presented in this study are available upon reasonable request from the corresponding author (Limei Chen).
